# Emerging Tools to Support DILI Assessment in Clinical Trials with Abnormal Baseline Serum Liver Tests or Pre-existing Liver Diseases

**DOI:** 10.1007/s40264-024-01511-8

**Published:** 2025-02-11

**Authors:** Jasmine Amirzadegan, Bereket Tesfaldet, Y. Veronica Pei, Eileen Navarro Almario, Mark I. Avigan, Paul H. Hayashi

**Affiliations:** 1 Center for Drug Evaluation and Research, Office of New Drugs, Division of Hepatology and Nutrition, Food and Drug Administration, White Oak, Silver Spring, MD USA; 2 Center for Drug Evaluation and Research, Office of New Drugs, Biomedical Informatics and Regulatory Review Science, Food and Drug Administration, White Oak, Silver Spring, MD USA; 3 Center for Food Safety and Applied Nutrition, Office of Analysis and Outreach, Biostatistics and Bioinformatics, Food and Drug Administration, White Oak, Silver Spring, MD USA; 4 Center for Drug Evaluation and Research, Office of Surveillance and Epidemiology, Office of Pharmacovigilance and Epidemiology, Food and Drug Administration, White Oak, Silver Spring, MD USA; 5 Oak Ridge Institute for Science and Education, Oak Ridge, TN USA

## Abstract

Based on the late Dr. Hyman Zimmerman’s observation that hepatocellular drug-induced liver injury (DILI) leading to jaundice carries a ≥ 10% fatality risk (coined as Hy’s law by others), evaluation of Drug-Induced Serious Hepatotoxicity (eDISH) continues to play a central role in the assessment of a study drug’s liability for acute hepatocellular DILI. The eDISH identifies drugs in clinical trials with DILI fatality (death or transplant) risk that may be unacceptable in a post-market setting. As a two-dimensional graph that plots peak total bilirubin (TB) versus peak serum aminotransferase levels for each patient during study drug or comparator treatment, eDISH identifies potential cases of acute, modest, and serious hepatocellular DILI for in-depth analysis of liver tests (LT) and clinical course so that the likelihood of causal association with the study drug can be determined. Unfortunately, the generalizable utility of this tool only pertains to trials enrolling patients with normal or near normal (NNN) baseline (BL) serum LTs. The eDISH does not necessarily apply to trials of patients with abnormal baseline (ABN-BL) LTs that often coincide with underlying liver disorders. Because drug development programs being reviewed by the FDA increasingly target liver disorders, we are often challenged to evaluate DILI risk in trials of patients with ABN-BL LTs. Also, the high background prevalence of metabolic dysfunction associated steatotic liver disease (MASLD) means patients with LTs above NNN may need to be enrolled in trials treating non-liver disorders to reflect the target population. Such study populations create challenges for industry and regulators because eDISH may not reliably categorize or identify potential cases of DILI for further analysis, as it so efficiently does in NNN-BL trials. We describe the main functionalities of eDISH in NNN-BL trials to understand what should be emulated by new tools or eDISH modifications. We then discuss non-eDISH–based plots that may be useful in ABN-BL trials.

## Key Points

**Table:** 

eDISH continues to be instrumental in detecting unacceptable DILI risk in clinical trials with normal or near normal baseline liver tests.
eDISH is not useful for clinical trials with markedly abnormal baseline liver tests.
New graphic tools using Sankey, composite, and waterfall plots may fulfill eDISH functionalities for trials with abnormal baseline liver tests

## Introduction

**Fig. 1:**
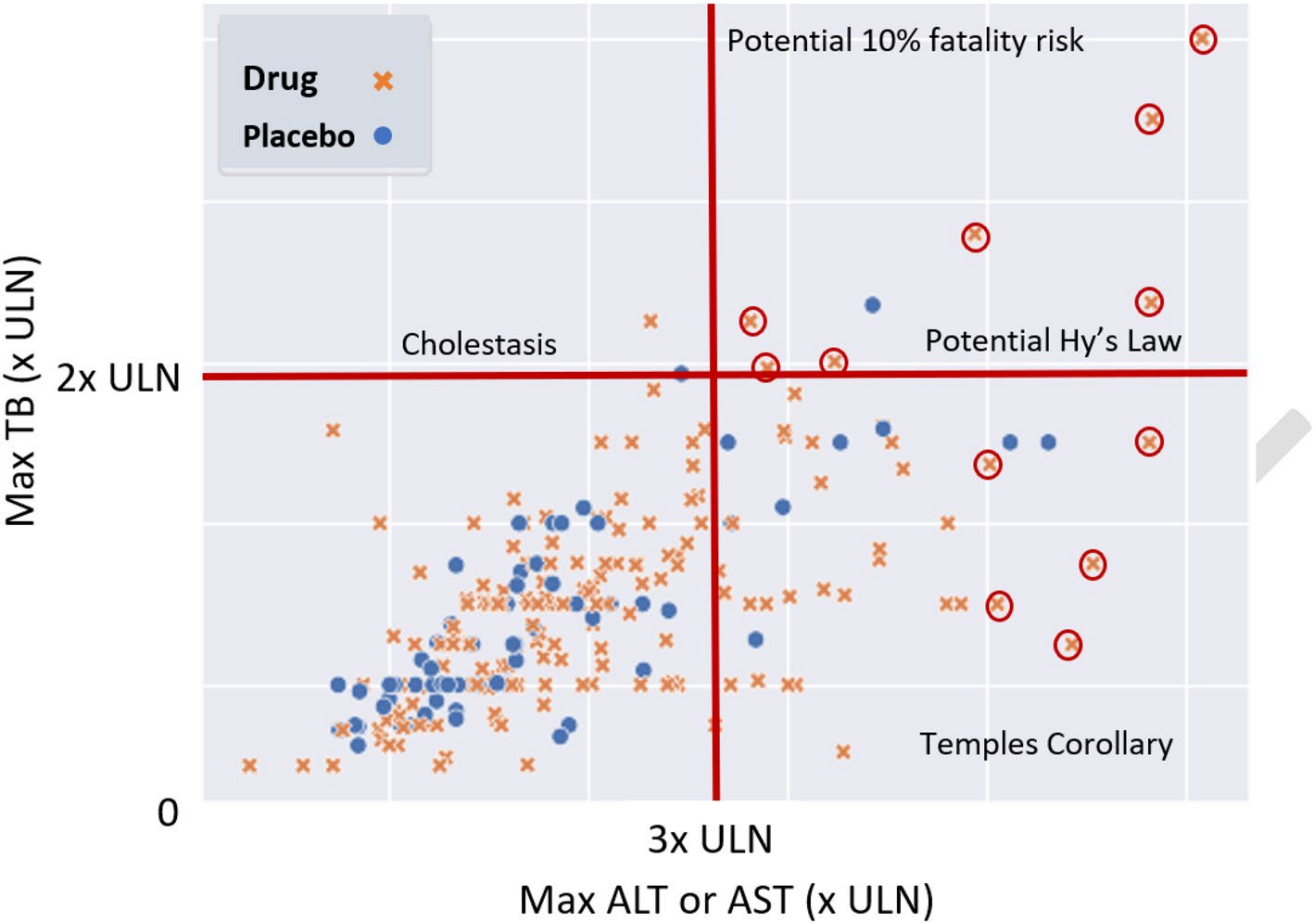
Hypothetical eDISH plot of a trial with active treatment (Drug x) and placebo arms, enrolling approximately two to one. Each subject’s maximum (Max), on-treatment total bilirubin (TB) and maximum on-treatment aminotransferase (alanine aminotransferase [ALT] or aspartate aminotransferase [AST]) are plotted. Four quadrant categories are defined by TB >2× upper limit of normal (ULN) and ALT or AST ≥ 3 × ULN. Potential Hy’s Law quadrant may contain drug-induced liver injury (DILI) subjects with a 10% fatality risk. The subjects circled in red may be prioritized for in-depth review

For nearly two decades drug developers and clinical scientists at the US Food and Drug Administration (FDA) have used evaluation of drug-induced serious hepatotoxicity (eDISH) as a screen to evaluate hepatocellular drug-induced liver injury (DILI) risk in clinical trials [1]. eDISH is a two-dimensional scatterplot displaying peak, on-treatment serum total bilirubin (TB) and aminotransferase (AT) values in multiples of upper limit of normal (×ULN) of each subject in active drug and comparator arms. It can identify all potential cases of hepatocellular DILI in the study population [2–5]. eDISH defines four quadrant categories by a horizontal line at TB 2×ULN and a vertical line at AT 3×ULN (Fig. 1). These four quadrants are important because they segregate subjects by DILI severity risk.

The late Dr. Hyman Zimmerman made the seminal observation that hepatocellular DILI leading to jaundice (TB >2×ULN) carries a 10% or greater risk of fatality (death or transplant). Coined by others as Hy’s Law, this observation was later confirmed in large observational studies [6–8]. In clinical trials, cases of acute hepatocellular DILI with consequent jaundice are identified by alanine aminotransferase (ALT) or aspartate aminotransferase (AST) > 3×ULN, TB > 2×ULN, no substantial alkaline phosphatase (ALP) elevation and no non-DILI cause found [9]. Such cases are considered to have a 10% or higher fatality risk consistent with Hy’s Law and plot to the right upper quadrant on eDISH. Just one or two Hy’s Law cases in a clinical trial study population are considered reliable predictors of substantial risk for hepatocellular DILI leading to fatalities in a larger post-market treatment population. Thus, cases meeting Hy’s Law serve as surrogate markers for rarer DILI fatalities that will occur at one-tenth the frequency of the Hy’s law cases observed in trials.

It is remarkable that since 2000, when broad adoption of eDISH by drug developers began, followed by release of an FDA guidance on the Premarketing Evaluation of DILI in Clinical Trials in 2009, no drugs have been removed from the market due to post-market cases of severe idiosyncratic hepatocellular DILI, compared to the late 1990s when several drugs with this liability were withdrawn.

Despite its ongoing success in trials enrolling subjects with normal or near normal (NNN) baseline (BL) liver tests, eDISH has substantial limitations when subjects have abnormal baseline (ABNL-BL) values. For the purposes of tool development, we considered NNN as ALT < 2× to 3×ULN and TB < 2×ULN, while we considered ABNL-BL as greater than those cut-offs. In NNN-BL trials, the pretreatment levels are all within a relatively narrow range. Therefore, on-treatment elevations are meaningfully compared between treatment arms in eDISH that plots values in × ULN. In contrast, the same × ULN boundaries may not meaningfully define potential DILI cases in ABN-BL trials. For example, it is unclear how to interpret DILI risk for a subject in a particular eDISH quadrant when they may have started in that quadrant before treatment. Indeed, ALT and TB values may have improved for such a subject, but such improvement will not be evident on eDISH. Erosion of the eDISH screening value is a growing concern as new drugs are increasingly developed to treat liver disorders with markedly ABN-BL liver tests (e.g., alcoholic hepatitis, biliary atresia, primary sclerosing cholangitis). Also, the high prevalence of metabolic dysfunction associated steatotic liver disease (MASLD) in the general population may mandate enrollment of subjects with ABNL-BL liver tests in trials targeting non-liver diseases (e.g., diabetes mellitus, hypertension, cancers) to reflect the target population. It will be difficult, if not impossible, to screen for DILI using eDISH in such studies. We describe the utilities of eDISH in NNN-BL trials to better understand what is needed from any modified eDISH or new screening tools for ABN-BL trials. We then present non-eDISH–based instruments that may fill these needs.

## What eDISH Does for NNN Trials and Not Do for ABN-BL Trials

Before modifying eDISH or creating other tools for ABN-BL trials, it is important to note three key filtering functions that eDISH fulfills in NNN-BL trials. These functions are (a) visualization of shift in liver tests between study arms, (b) categorization of subjects into groups of different liver injury severity, and (c) efficient identification of individual subjects with potential DILI for priority assessment ideally with point-click linkages to case-level data [1]. Any new or modified system for ABN-BL clinical trials will likely need to demonstrate all three functions to have utility similar to eDISH in NNN trials.

As described above, eDISH plots each subject’s peak on-treatment ALT and TB values in × ULN (Fig. 1). Fulfilling the first function, one can visually compare broad positioning of the plotted data between the study drug and comparator arms. The eDISH then sets boundaries for elevated TB of > 2×ULN (i.e., jaundice) and ALTs at ≥ 3×ULN, thereby defining four quadrants. Subjects with peak values in the upper right, or Potential Hy’s Law quadrant may have hepatocellular DILI carrying a 10% or higher fatality risk if the ALT and TB changes are due to DILI, and alkaline phosphatase (ALP) is not greatly elevated. Those in the right lower, or Temple’s Corollary quadrant had ALT elevations but did not develop jaundice. Proportionately more subjects on study drug in this quadrant support concern about DILI cases in the Potential Hy’s Law quadrant. Subjects in the left upper quadrant had jaundice but no substantial ALT elevation, suggesting possible cholestatic injury. Thus, these quadrant groups represent meaningful categories. Finally, one can quickly identify subjects with the more severe liver injury to prioritize for in-depth review (Fig. 1, red circles). The utilities of these three functions (shift of tests, categorization, and review prioritization), which eDISH provides so simply and efficiently in NNN-BL trials, and the difficulties created by their loss in ABN-BL trials cannot be over stated.

**Fig. 2:**
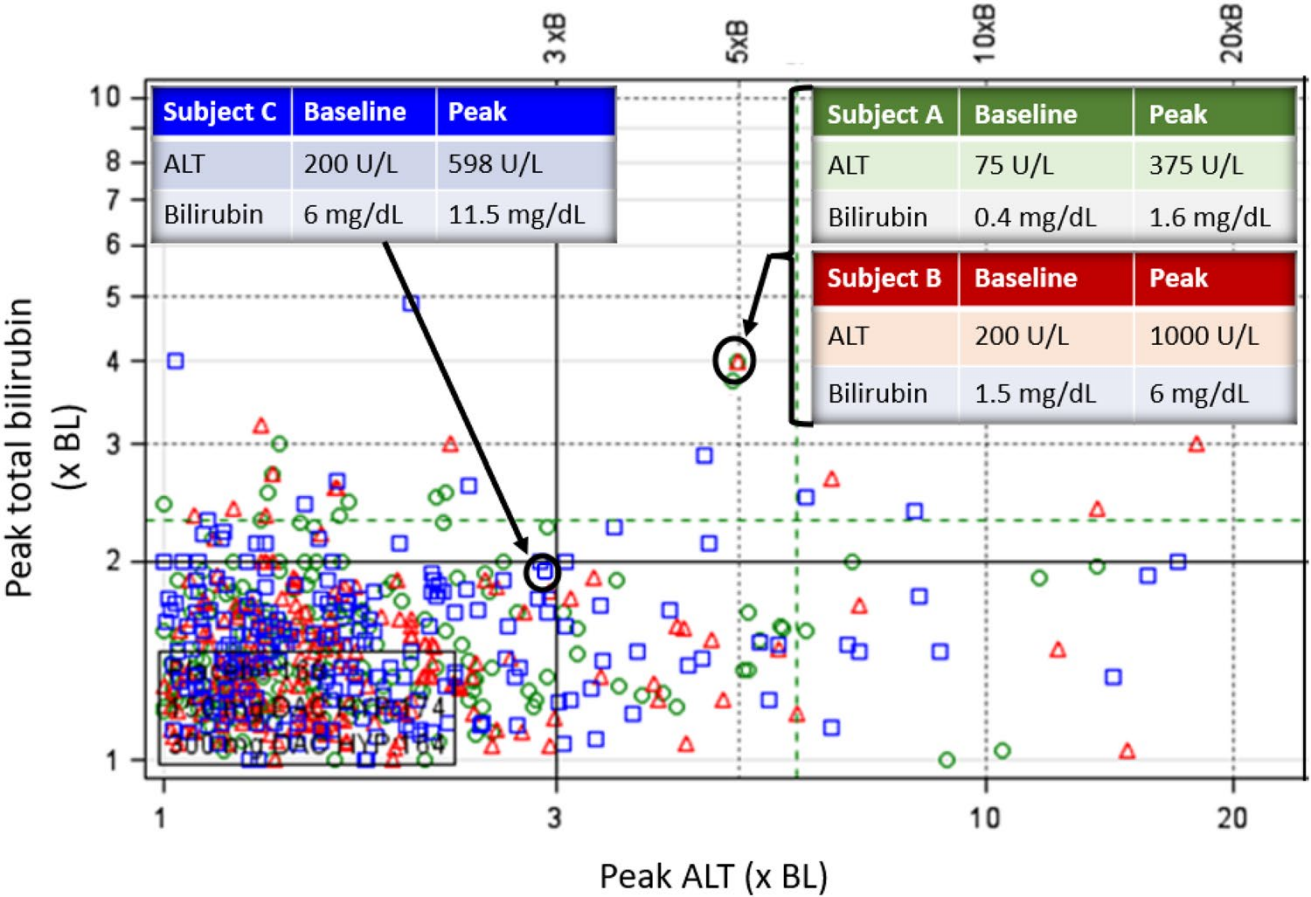
Modified eDISH (mDISH) plot based on peak total bilirubin (TB) and peak alanine aminotransferase (ALT) in times baseline (× baseline [BL]) and modified from a clinical trial with three treatment arms (red triangles, blue squares, and green circles). Each data point represents one subject’s peak on-treatment TB and ALT levels in multiples of their own baseline values. Hypothetical Subjects A and B plot to the same location because each had a peak bilirubin four times their baseline values and peak ALT five times their baseline values. However, Subject B’s jaundice and ALT of 1000 U/L suggest a more severe potential drug-induced liver injury (DILI) compared to Subject A’s bilirubin and ALT. Subject C’s deeper jaundice and ALT rise suggest a more worrisome injury compared to Subject A’s or B’s bilirubin and ALT, yet Subject C is lower and more to the left compared to Subjects A and B

As a screening tool, eDISH cannot fulfill these three functions in ABN-BL trials because each subject’s on-treatment plot location is no longer anchored to a NNN baseline. From an eDISH plot, it is impossible to tell whether subjects that meet peak liver test criteria for Hy’s Law during treatment had these LT abnormalities already at baseline. In fact, some may have improved with decline in TB and ALTs from even higher baseline levels. eDISH will not graphically reflect declines in such cases. The same is true of the Temple’s Corollary and left upper quadrant categories. Thus, visualization of shift, categorization, and identification of subjects with new-onset severe liver injury associated with the study drug will not be readily accomplished using eDISH alone. The eDISH modified to plot each subject’s peak ALT and TB in multiples of their own baseline (×BL), also known as a modified eDISH (mDISH), may only capture the first eDISH function: visualization of shift from baseline between study arms. Because each subject’s baseline can be quite different, one cannot separate jaundiced from non-jaundiced subjects or subjects with towering ALTs from those with only modest ALTs during treatment. Therefore, meaningful categorization and subject identification with mDISH are not accomplished. In Fig. 2, hypothetical subjects A and B plot to the same point on mDISH because they had the same × BL changes, yet their absolute values are quite different. Subject B is jaundiced with an ALT reaching 1000 U/L and should have a higher categorization and prioritization for DILI assessment compared to Subject A, yet they do not separate on mDISH. Subject C had jaundice and modest ALT elevation at baseline, typical of patients with alcoholic hepatitis or biliary atresia. The ALT and TB nearly tripled and doubled, respectively. Arguably, Subject C may have the highest risk of DILI fatality, yet plots substantially lower and to the left of Subjects A and B.

Therefore, mDISH alone can only provide visualization of shift in liver tests from individual BLs. Indeed, categorization using boundaries of 3×BL ALT and 2×BL TB as threshold criteria for potential DILI cases should not be placed on mDISH as they do not provide discriminatory value, nor should the quadrant labels of Hy’s Law, Temple’s or Cholestasis be used. There are no such equivalent quadrants on mDISH that distinguish levels of acute liver injury severity that may be associated with increased fatality. Furthermore, attempts to apply Hy’s Law fatality risk using any tool should be done with caution in ABN-BL trials. The DILI-related fatality may be difficult to discriminate from other complications of underlying hepatic disease, and liver diseases likely have widely different tolerances of DILI. Hy’s Law cases would first need to be defined for chronic liver diseases, which may be quite challenging due to the diversity of these diseases with variable ABN-BL values and fibrosis stages. Such definitions would then need further study to verify a reliable percent mortality rate. To our knowledge, neither of these tasks have been done for even one chronic liver disease.

Schneider et al used eDISH, mDISH, and an hDISH, which plotted changes above BL in × ULN for an Alagille’s trial [10]. While these plots provide useful data, it is unclear how to easily identify subjects with highest absolute values on hDISH, and the complexity of using three different plots makes this method less useful for quick visualization of shift and categorization. Similarly, the Council for International Organizations of Medical Sciences (CIOMS) consensus report on DILI suggests side-by-side display of eDISH and mDISH may help “put outlier cases in perspective,” but how to efficiently categorize and prioritize cases is not stated [5]. Presumably these two functions would have to be done one case at a time. Parks and colleagues reset the cut-offs defining the four eDISH quadrants for oncology trials based on aggregated trial data [11]. It is unclear whether these cut-offs categorize subjects into meaningful DILI mortality risk, and data are limited to oncology trials. To our knowledge, no eDISH modifications have reproduced the three functions in a quick and easy-to-use manner that mimics eDISH in NNN-BL trials.

## Sankey Plotting

The missing critical variable in eDISH and mDISH is where subjects start before treatment with study drug in terms of absolute values of ALT and TB (i.e., × ULN). The challenge is adding these baseline values to two-dimensional plots that have the two axes already used for peak ALT and TB values. Three-dimensional or interactive plots lack the simplicity of eDISH creating complicated data visualization. Therefore, we assigned one of four quadrant categories of eDISH to each subject (a) at baseline and (b) on-treatment based on peak values. Both baseline and on-treatment categorization used ALT and TB values in × ULN. By these assignments, we accomplished two things: (1) we collapsed the data to just two categorical variables for two-dimensional Sankey plotting, and (2) we re-anchored the data on absolute values in × ULN that was so successful for eDISH, rather than × BL values that vary by subject. We beta tested these plots on two trials treating two different chronic liver diseases.

### Methods

**Fig. 3:**
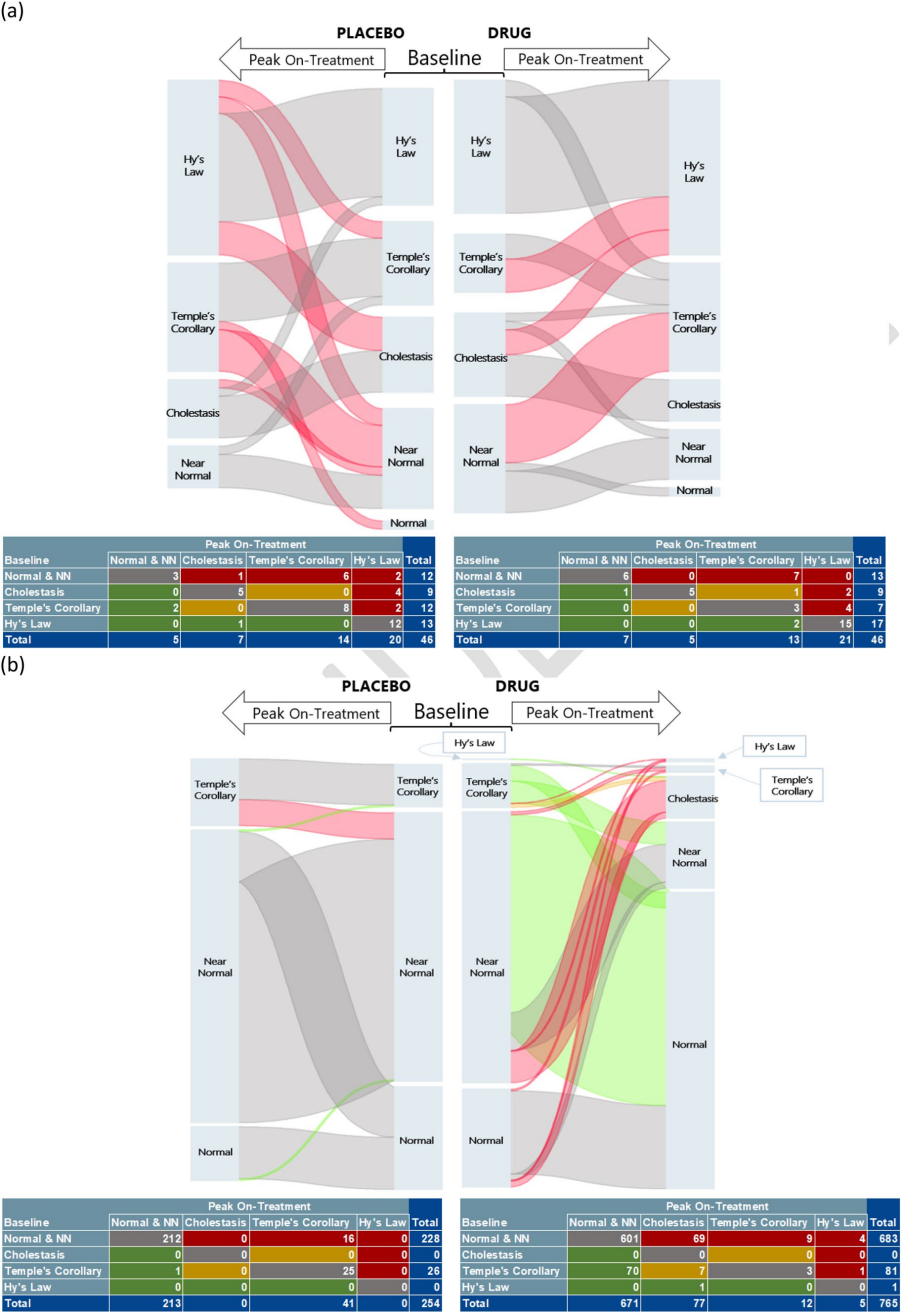
Sankey plots and cross table counts for **a** Study 1 and **b** Study 2. Sankey baseline categorizations are in the middle. Peak on-treatment shifts from baseline for the placebo treatment arm move left, while shifts from baseline for the active treatment arm move right. All categorizations are based on × upper limit of normal (ULN) alanine aminotransferase (ALT) and total bilirubin (TB) levels. Unfavorable shifts (potential drug-induced liver injury [DILI]) move upward and are highlighted in pink. Study 1 had fewer types of upward shifts (3 vs 5), and fewer total numbers in these shifts (13 vs 15) in the drug arm compared to placebo. Study 2 had markedly more upward (pink) and downward (green) shifts in the drug arm compared to placebo arm. Cross tables have shift counts with unfavorable (potential DILI) in red, favorable (potential efficacy) in green, and neutral shifts in grey and yellow for each arm. Near normal (NN) = ALT between ULN and < 3 × ULN

We used R version 4.3.3 with “dplyr”, “tidyr”, “ggplot2”, “ggsankeyfier”, “stringr”, “magick”, and “kableExtra” libraries. We classified all subjects into four baseline categories prior to treatment based on eDISH cut-offs of ALT ≥ 3 × ULN and TB >2 × ULN. We oriented the Sankey plots such that the baseline categories were hierarchically placed for all study subjects in the center of the figure with the most severe category (ALT and TB qualifying for Hy’s Law quadrant) at the top. Subject movements to their on-treatment peak ALT and TB category are shown as shifts leftward from their baseline for subjects receiving placebo and rightward for subjects receiving active drug (Fig. 3). Shifts of concern (potential DILI) were indicated by a move upward in severity category during treatment from baseline (e.g., NNN at baseline to Hy’s Law category on treatment) while shifts of improvement move downward (e.g., Temple’s quadrant at baseline to NNN quadrant on treatment). We made upward shifts of concern pink for both studies and added green highlighting for downward shift for Study 2. Thus, the plots were oriented to give side-by-side visualization of shifts between study arms by comparing the thicknesses (proportions) of different upward shifts defined by ALT and TB changes. Cross tables first used by Tesfaldet et al to catalog changes in eDISH quadrant assignments [12], provide numeric tallies of each shift with top and rightward entries in red representing concerning upward shifts (potential DILI), bottom and leftward, green boxes suggesting improvement (potential efficacy), and grey and yellow boxes as potential neutral shifts.

### Results

Study 1: In this trial, 92 subjects with a severe chronic liver disease (CLD) were randomized 1:1, placebo to study drug. Sankey plots (Fig. 3a) provide visualization of on-treatment shifts between the four categories typically defined by eDISH. The plots for the placebo and active treatment arms are side-by-side with baseline categorization in the middle to facilitate comparisons of baseline randomization and shifts of interest. Corresponding cross table counts are below each Sankey plot. Study 1 subjects had markedly elevated pre-treatment baseline ALT and TB levels with most subjects not in the NNN category, and large proportions of subjects qualifying for Hy’s Law quadrant of an eDISH at baseline (28%, 13 of 46, placebo arm; 37%, 17 of 46, active drug arm). During treatment, there were three upward on-treatment shifts, highlighted in pink, based on worsening peak liver test categories in the active drug arm compared with five such upward shifts in the placebo arm. While two of the three upward shifts on drug were thicker indicating higher proportions moving on this path compared to placebo, the overall cross-table tally for all pink shifts of concern was higher in the placebo arm (15 vs 13) (Fig. 3a). Notably, liver test results from no subject in the active arm during treatment shifted to the Hy’s Law quadrant from NNN, but two placebo subjects shifted along this path representing the most marked upward shift. In this trial of patients with CLD, the Sankey plots provided visualization of treatment-associated shifts in ALT and TB concerning for DILI between active treatment and placebo. The shifts are tallied by cross-table data showing the numbers of cases undergoing each shift type. These analytic tools for CLD trials have utilities that are comparable with eDISH graphs for NNN trials. In the active drug arm of CLD trials, cases in upward pink shifts of Sankey plots may include potential DILI cases that require in-depth evaluation, analogous to cases contained in the Hy’s Law, Temple’s Corollary, and left upper quadrants of eDISH for NNN trials.

Study 2: This trial treated 1019 patients with a CLD, randomized 1:3, placebo to study drug (active arm). Unlike Study 1, most subjects were in the NNN category at BL, but 26 of 254 (10%) placebo subjects and 81 of 765 (11%) active arm subjects had liver tests that would put them in the Temple’s Corollary quadrant of eDISH at baseline. Unlike Study 1, there were stark shift differences between treatment arms (Fig. 3b). There was very little improvement (downward shift) with some cases shifting to more serious liver test categories in the placebo group that may reflect disease progression, compared to substantial downward shifts (improvement) highlighted in green in the active treatment arm. The active treatment arm also had several upward shifts of concern, highlighted pink, marked by worsening ALT and/or TB during treatment not seen in the placebo arm. Cross-tables provide case numbers in each shift subset. Again, these are grouped by color, with the subsets of patients that shift to more serious liver test categories during treatment in red, versus those with improved test categories in green. The shifts toward worsening categories represent subsets in which cases of potential DILI should be flagged for in-depth assessment.

## Composite Plotting

**Fig. 4:**
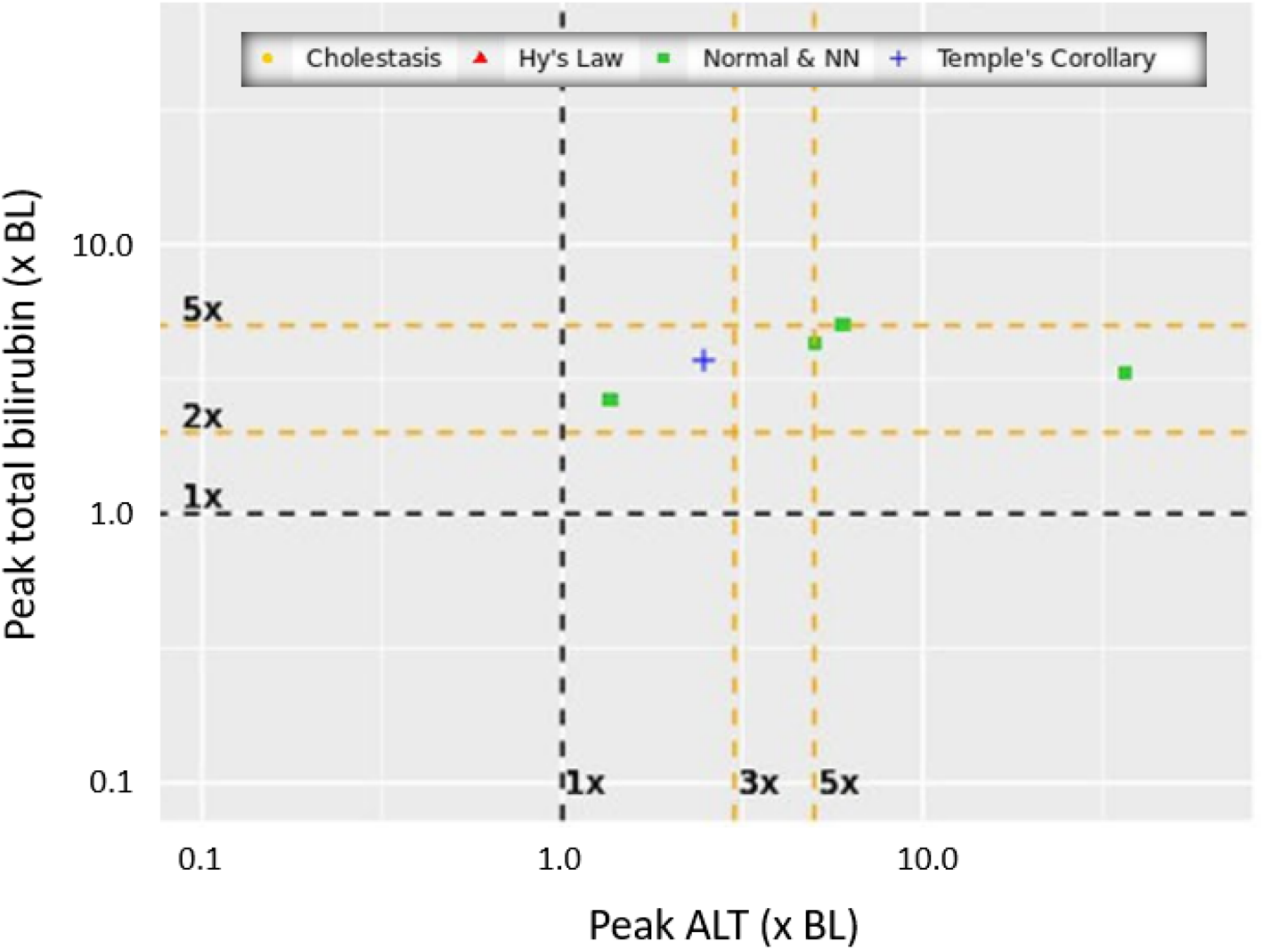
Composite plot modified eDISH (mDISH) plot adapted from Tesfaldet et al [12]. Only the five subjects from Study 2 who met alanine  aminotransferase (ALT) (× upper limit of normal [ULN]) and total bilirubin (TB) (×ULN) criteria for Hy’s Law quadrant are plotted. The category (quadrant) of origin for each subject is indicated by marker color and shape. Four of the five subjects started in the normal or near normal quadrant at baseline (green squares), and one subject started in Temple’s quadrant at baseline (blue plus sign). There were no subjects in Hy’s Law quadrant based on peak values who were in that quadrant at baseline or in the cholestasis quadrant at baseline

The Sankey plots are not able to prioritize individual subjects for case assessment within each shift subset because they do not plot patient-level ALT and TB values in absolute units, × ULN, or × BL. Therefore, to complete the third eDISH functionality, we used composite plotting described by Tesfaldet et al [12]. That publication also analyzed Study 2 data, and liver test results are displayed in four distinct mDISH plots, to focus on each severity category (NNN, cholestasis, Temple’s, and Hy’s Law) based on each subject’s on-treatment peak level ALT and TB levels. In Fig. 4, we show one of their published mDISH that plots × BL values only for the subjects that qualify to be in a typical eDISH Hy’s Law quadrant using on-treatment peak liver test results. The plot identifies each subject’s category of pre-treatment origin by a distinct color and symbol. In this case, all five subjects were new arrivals in the Hy’s Law category, four shifting from a NNN category and one from a Temple’s category, as previously shown in the Fig. 3b cross-table. Had a subject met Hy’s Law by baseline and on-treatment ALT and TB criteria (i.e., no category shift), there would be a red star on this mDISH plot. The shifts of NNN to Hy’s Law and Temple’s to Hy’s Law are two of the categories of concern identified in the Sankey plot, which are then captured for case level analyses in the composite plot for subjects ending up in Hy’s Law. Similar composite plots for subjects ending up in the three other on-treatment categories of concern were also created allowing complete subject identification, as needed [12].

## Modified Waterfall Plot

Study 2 had only one subject with baseline jaundice likely due to inclusion and exclusion criteria. Such limitation on baseline bilirubin allowed exploration of a modified waterfall plot, using only baseline and maximum on-treatment ALT values measured in absolute U/L. For this plot, we used Python version 3.12 with the “pandas” version 2.2.1 library, and R version 4.3.3, with the following libraries: “dplyr”, “kableExtra”, “ggplot2”, “gridExtra”, and “magick.” These plots have been used in other publications, but are not formally named [13, 14]. To distinguish them from typical waterfall plots that place baseline on a horizontal line, we refer to them as modified waterfall plots.

### Methods

**Fig. 5:**
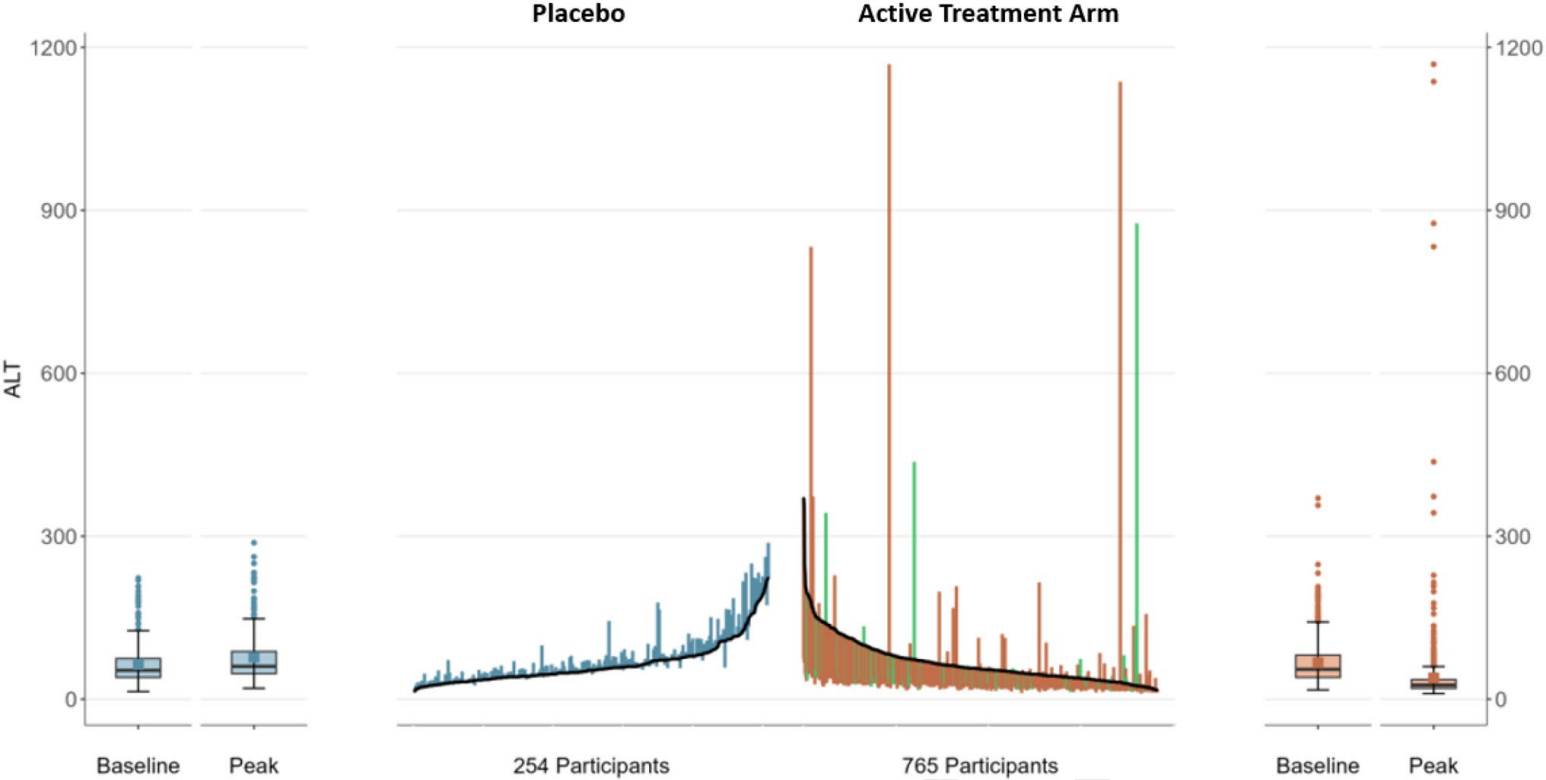
Modified waterfall plot for Study 2. Alanine aminotransferase (ALT) levels in U/L are plotted for each subject’s baseline value along the black lines and each subject’s maximum change on treatment are depicted by vertical bars, blue for the placebo arm subjects and bronze for active treatment arm subjects. Subjects who developed jaundice have green bars. The active treatment arm had more subjects with decrease in ALT seen by the large number of bronze bars dropping below baseline compared to the number of blue bars dropping below baseline for placebo subjects. However, there were more subjects in the active treatment arm with substantial increases in ALT over baseline and several developed jaundice. These cases may have had drug-induced liver injury (DILI) and can be easily identified and prioritized for in-depth analyses

This modified waterfall plot has placebo on the left and active arm on the right (Fig. 5). Absolute on-treatment ALT in U/L is scaled along the vertical axes. Subjects are then ordered along the horizontal axis by baseline ALT value. Placebo subjects are ranked lowest to highest and active treatment arm subjects highest to lowest moving left to right creating the black lines with highest baseline values in the middle of the plot. Each subject’s maximum ALT increase or decrease is shown for the placebo and active treatment arm subjects in blue and bronze bars, respectively. Summary box, whisker, and outlier plots are on the far left and right. Cases with new onset jaundice are identified as a green bar in the active treatment arm.

### Results

The variable baseline or modified waterfall plot provides visualization of baseline ALT values and shift in ALT between study arms with significantly higher ALT increases relative to baseline in the active treatment arm compared to placebo, which showed only modest changes (Fig. 5). Improvement in ALT on study drug is easily seen by the numerous bronze bars dipping below baseline levels in the drug treatment group, whereas those with potential DILI show increases above their baselines. Categorization of those cases with potential DILI by different levels of severity can be made by setting horizontal ALT cut-offs (e.g., over 300, 600, or 900 U/L) with and without jaundice (green bars). Individual cases with particularly severe liver injury can be easily identified by the amount of ALT increase from baseline and whether the bar is green (new jaundice). Such cases can then be prioritized for case level assessment and adjudication. The active arm in this trial used a drug that is associated with hemolysis, which may explain the jaundice in some subjects with maximum change in ALT being below baseline.

## Discussion

The inability for eDISH to function properly in clinical trials in which treated subjects have abnormal baseline liver tests (ABN-BL trials) creates a substantial challenge for DILI assessment in drug development. Thus far, eDISH modifications have not reproduced the three functions so elegantly provided by eDISH in NNN-BL trial analyses. We submit that these three functions, (a) visualization of liver test shift, (b) categorization of subjects by severity, and (c) identification of subjects for detailed assessment, should be reproduced in any new DILI screening tools for ABN-BL trials. Furthermore, any new tool should be as simple and as intuitive as possible.

Sankey plots display shifts in subjects from their baseline to their on-treatment eDISH category based on ALT and TB in multiples of upper limit of normal (×ULN). The plot, and accompanying cross table of shift counts, provide visualization of shifts between treatment arms and shift categories with higher potential severity of liver injury, thus meeting the first two eDISH functionalities in an easy-to-understand manner. The cross tables provide counts for each shift category similar to the four quadrants category counts that accompany eDISH. The use of category proportions in Sankey plots has an additional advantage over eDISH or mDISH in trials with markedly unequal randomization. An eDISH or mDISH for Study 2 would have three times as many of the active treatment arm subjects plotted compared to placebo. Such unequal numbers in each arm on scatterplots can make shifts and quadrant imbalances between arms difficult to see. To accomplish the third eDISH function of subject identification, we used composite plotting of subjects by on-treatment category (i.e., quadrant to which subjects  shift) published by Tesfaldet et al [12]. Their composite plotting uses × BL (mDISH). By this two-step process of Sankey plotting with cross table followed by composite plots, all three original eDISH functionalities are met. This method is not as elegant nor as quick as eDISH, but we contend such simplicity may not be possible given the necessity of accounting for baseline status in ABN-BL trials.

For trials that exclude subjects with jaundice, a modified waterfall plot may provide all three functionalities in one figure, approaching the simplicity of eDISH in NNN-BL trials. Such a plot is possible because the TB at baseline is in the NNN range for all subjects. This plot may be particularly useful in MASLD trials that exclude subjects with jaundice (e.g., subjects without cirrhosis). In fact, when to use the Sankeys and cross tables with composite plots versus modified waterfall plots may depend on whether and by how much both ALT and TB are elevated at baseline as well as the underlying liver disease. For example, the Sankey and cross table have the ability to add a category of ALT >3 × ULN and TB between 1.5 and 2×ULN for finer resolution of patient shifts in certain liver disease sub-populations. We provide starting point suggestions for when to apply the plots based on baseline ALT and TB in Table 1. We intend to put our codebases on the GitHub repository for others to try. The composite plot code is already accessible via https://github.com/FDA/Composite-eDish-Plot/.

**Table 1:** Potential applicability of Sankey-cross tables with composite plots versus modified waterfall plotting by degree and type of abnormal baseline liver tests

Baseline ALT and TB levels in a substantial proportion of studied population	Sankey-cross table, with composite plotting	Modified waterfall
ALT ≥ 3×ULN TB >2×ULN (e.g., alcoholic hepatitis, biliary atresia)	++	−
ALT ≥ 3×ULN TB ≤1.5×ULN (e.g., non-cirrhotic chronic liver diseases with low TB)	+	++
ALT ≥ 3×ULN TB 1.5 to 2×ULN (e.g., chronic liver disease with early cirrhosis)	++	+
ALT < 3×ULN TB >2×ULN (e.g., stable, or quiescent chronic liver disease with cirrhosis)	++	−

Positive signs suggest applicability. Negative signs suggest inapplicability

*ALT* alanine aminotransferase, *TB* total bilirubin, *ULN* upper limit of normal

These plots and tables have limitations. First, subjects who stay in Hy’s Law or Temple’s categories throughout a trial may worsen greatly with ALT and/or TB increases. Thus, they cannot be summarily ignored on a Sankey and need examination on composite plotting. Second, our modified waterfall plot uses the maximum absolute ALT change from baseline, be it increase or decrease. While such plotting captures potential efficacy, it may miss DILI cases that occur when an ALT increase is less than the subject’s absolute on-treatment decline. We plan to construct modified waterfalls of only maximum increases in ALT, thereby focusing on DILI risk. Third, we have only studied two chronic liver disease trials and thus have limited experience across the array of liver diseases being targeted. Other liver disease trials may be less amenable to our plot designs. Fourth, the plots do not address cholestatic DILI. We are planning analogous plotting using ALP instead of ALT, but the utility of such efforts is unclear. Finally, like any tool for ABN-BL trials there are no validated fatality rates that correspond to any Sankey plot, cross table, or modified waterfall plot groupings similar to those associated with the Hy’s Law quadrant for hepatocellular DILI in NNN-BL trials. Until such fatality rates in ABN-BL trials are established for defined liver disorders, DILI assessment cannot be based on risk thresholds like those defined by Hy’s Law and Temple’s Corollary in NNN-BL trials, regardless of the tools used. All these limitations may be particularly problematic for trials targeting the various stages of alcohol-related liver disease, which have heterogenous baseline liver tests that can fluctuate widely.

The ABN-BL trials for chronic and acute liver diseases are generating large amounts of data that will require a different DILI screening approach. Neither eDISH nor modified eDISH plots alone will be adequate. A two-step process with Sankey plots and cross-tables, followed by composite plotting, or a single modified waterfall plot if hyperbilirubinemia is restricted at enrollment, may recapture the three functions that eDISH provides in NNN-BL trials. These and other tools should be explored further, particularly in trials with baseline liver enzymes well over three to five times ULN and/or baseline jaundice. Accurate DILI assessment will be challenging, but these trials will provide valuable information on whether such tools can be useful.

## Declarations

### Disclaimer

The conclusions and views in this manuscript are the authors and do not represent an official position of the Food and Drug Administration.

### Availability of Data

Not applicable. Datasets from new drug applications are not publicly available.

### Code Availability

Composite coding available on GitHub. Other coding to be available on GitHub shortly.
